# Next-generation sequencing in breast cancer: current clinical applications and future directions

**DOI:** 10.1080/07853890.2025.2569989

**Published:** 2025-10-06

**Authors:** Nur Fatihah Mohd Zuhdi, Alaa Siddig, Siti Norasikin Mohd Nafi, Md Salzihan Md Salleh, Maya Mazuwin Yahya, Wan Zainira Wan Zain, Tengku Ahmad Damitri Al-Astani Tengku Din, Wan Faiziah Wan Abdul Rahman

**Affiliations:** ^a^ Department of Pathology, School of Medical Sciences, Universiti Sains Malaysia, Kelantan, Malaysia; ^b^ Department of Biomedical Sciences, College of Health Sciences, Abu Dhabi University, Abu Dhabi, United Arab Emirates; ^c^ Department of Surgery, School of Medical Sciences, Universiti Sains Malaysia, Kelantan, Malaysia; ^d^ Breast Cancer Awareness and Research Unit, Hospital Pakar Universiti Sains Malaysia, Kelantan, Malaysia; ^e^ Department of Chemical Pathology, School of Medical Sciences, Universiti Sains Malaysia, Kelantan, Malaysia

**Keywords:** Next generation sequencing, breast cancer, circulating tumour DNA, single cell RNA sequencing, HER-2 low breast cancer

## Abstract

**Introduction:**

Breast cancer is a heterogeneous disease that claims 670,000 lives by 2022. Omic technologies, particularly next generation sequencing (NGS) offers promising avenues for precision medicine. American Society of Clinical Oncology (ASCO) outlines genomic testing’s utility, emphasizing prognostic and diagnostic potential.

**Objectives:**

This review succinctly explores NGS’s evolution and clinical applications of NGS in breast cancer, thereby guiding future research to enhance patient care.

**Methods:**

Comprehensive literature searches were conducted using databases such as PubMed, Google Scholar, and ResearchGate, focusing on keywords including breast cancer, *HER-2 low breast cancer, circulating tumour DNA, single-cell RNA sequencing, and next-generation sequencing*. Peer-reviewed, high-quality articles published in English were selected for inclusion.

**Results:**

Previous studies have explored the evolution of NGS technology and its clinical applications in breast cancer, including genomic and transcriptomic characterization, treatment guidance, and resistance prediction. Molecular profiling of challenging entities such as early-onset breast cancer and HER-2 low tumours was summarized, with key findings highlighted. This review also discusses emerging technologies, including circulating DNA and single-cell sequencing, as promising avenues for discovery.

**Conclusion:**

NGS has revealed the genomic and transcriptomic diversity of breast cancer, identifying actionable alterations associated with chemotherapy response and resistance to therapies such as trastuzumab, TKIs, and CDK4/6 inhibitors. Circulating tumour DNA (ctDNA) shows potential for diagnosis, prediction, prognosis, and monitoring, despite tumour heterogeneity. Single-cell analysis enables exploration of individual cell transcriptomes, though high costs and low throughput remain barriers to widespread adoption. HER2-low tumours continue to pose significant research challenges.

## KEY MESSAGES

1. Next-generation sequencing plays a pivotal role in characterizing the genomic landscape of breast cancer, including diagnosis, prognosis, treatment selection, patient monitoring, and identification of actionable alterations associated with chemotherapy response and resistance to targeted therapies.
2. The HER2-low breast cancer subtype poses a challenge for the standard molecular classification of HER-2 status. NGS helps redefine this distinct molecular subtype by characterizing its underlying genomic signatures.
3. Emerging advances in NGS, such as single-cell RNA sequencing (scRNA-seq) and circulating tumour DNA assessment, hold promise for advancing the current understanding of breast cancer heterogeneity and treatment response.

## 1. Background

Breast cancer is a heterogeneous disease affecting 2.3 million women worldwide, with a high mortality rate accounting for 670,000 fatalities by 2022 [1]. Owing to recent breakthroughs in omics technologies, a more accurate approach to precision medicine is now feasible [2,3]. Next-generation sequencing (NGS) has been extensively utilized for diagnosis, prognosis, treatment selection, and monitoring of patients [4]. The American Society of Clinical Oncology (ASCO) has published an outline of the proper use of genomic testing in advanced or metastatic solid tumours based on clinical opinions from their expert panels and has acknowledged that genome sequencing could also yield prognostic or diagnostic indications [5]. Given that most of the existing NGS testing standards are Europe-centric, the Asia-Pacific Oncology Drug Development Consortium (APODDC) has released guidelines for using NGS in patients with metastatic cancers, specifically tailored to Asian patients [6]. Furthermore, with NGS, a pathologist can effectively combine molecular profiles and morphological changes when faced with challenging breast malignant cases [7].

This review offers a comprehensive summary of the current clinical applications of NGS in breast cancer and the future directions for this powerful tool. Even though conventional clinical markers like histology and immunohistochemistry aid in therapy, they frequently fall short in capturing the entire genetic complexity of cancers, including intratumoral heterogeneity and uncommon mutations that can be affected. Moreover, notwithstanding progress in targeted medicines, significant variation in tumour biology and treatment response remains. NGS helps address these gaps by making it possible to precisely characterize the genetic landscape, predict therapeutic response, create individualized treatment plans, and track the course of the disease using single-cell and circulating tumour DNA analysis. However, the translation of genetic data into therapeutic decisions depends on efficient collaboration among healthcare providers. A multidisciplinary team (MDT) of molecular pathologists, oncologists, bioinformaticians, genetic counsellors, and laboratory scientists is crucial for optimal test selection, precise data interpretation, and the incorporation of findings into patient care. This collaboration enhances diagnosis accuracy, informs treatment choices, and facilitates patient guidance/counselling, connecting sequencing data to better clinical results. This study, which synthesizes the current available data, paves the way for the identification of clinical application gaps in the literature and providing a reference for further research exploring how NGS may improve breast cancer patient care.

## 2. Genomic profiling technologies in breast cancer

### 2.1. Next generation sequencing (NGS) technology

Different types of NGS can be categorized based on the technology used or the targeted application or purpose of the experiment. More than three decades have passed since the invention of DNA sequencing technology in 1977. Subsequent advancements in sequencing platforms have resulted in substantial improvements, each contributing significantly to the advancement of genome research, clinical disease investigation, and pharmaceutical development. In this section, we briefly describe our evaluation of this technology.

#### 2.1.1. Sanger sequencing or first-generation sequencing

Sanger sequencing, alternatively called the first generation NGS, which was documented in 1977, is a modification of the Maxam-Gilbert chemical cleavage method to reduce the use of toxic reagents. Sanger sequencing utilizes specific nucleotides called dideoxy nucleotides, which lack a 3′-OH group. This prevents the formation of a phosphodiester bond by the DNA polymerase, leading to the termination of DNA chain growth at that particular position. These dideoxy nucleotides, labelled with radioactivity or fluorescence, facilitate detection in sequencing gels or automated sequencing machines. The Sanger sequencing by synthesis (SBS) dideoxy method is a widely accepted standard for DNA sequencing [8].

#### 2.1.2. Second-generation sequencing or massively parallel sequencing

According to the National Cancer Institute dictionary, massive parallel sequencing (MPS) is a high-throughput method used to determine a portion of the nucleotide sequence of an individual’s genome. This technique uses DNA sequencing technologies that are capable of processing multiple DNA sequences in parallel. This technique includes whole-genome sequencing (WGS), whole-exome sequencing (WES) and targeted gene panel. In MPS, millions of sequencing processes are conducted simultaneously, and the preparation of the DNA fragments is carried out through full automation; in certain cases, these features significantly decrease the expense and markedly boost the efficiency of genomic sequencing, overcoming the limitations of Sanger sequencing. Different platforms for MPS are available on the market, including Illumina, SOLiD, Roche GS-FLX 454 Genome Sequencer, Helicos, Pacific Biosciences, and nanopore sequencing. All of these platforms perform MPS; however, the different platforms have different sequencing chemistry, amplification methods, read length, turnaround time, and cost. In addition to different accuracies. The most common advantage of MPS is that a substantial number of germline and somatic mutations have been discovered. On the other hand, MPS, the beginning of the invention of this technology, suffers from several limitations, including difficulties in assembling short reads when compared with Sanger sequencing and the raw accuracy rate required to be reduced [9].

#### 2.1.3. Third-generation sequencing/long read sequencing

Long-read sequencers provide reads with lengths of more than 10 kb compared to short-read sequencing technologies, which provide reads with lengths of up to 600 bases. The advantage of long reads over short reads is that the assembly and mapping process will be easier. In addition, the recognition of structural variants will consequently improve. Third-generation sequencing provides more accurate and cost-effective sequencing options. Currently, common platforms available in the market provide long-read sequencing, including nanopore sequencing and PacBio Single-Molecule Real-Time (SMRT) sequencing [10].

In conclusion, the evolution of DNA sequencing technologies from Sanger sequencing to second-generation sequencing (SGS) and third-generation sequencing (TGS) has revolutionized genomic research and clinical diagnostics. Sanger sequencing, a pioneer in DNA sequencing, laid the foundation for modern sequencing methods by introducing the concept of chain termination. SGS, also known as Massively Parallel Sequencing (MPS), enhances throughput and reduces costs by enabling the simultaneous processing of millions of DNA fragments. Despite its initial limitations, MPS overcame the challenges associated with Sanger sequencing and became a cornerstone in genomics, facilitating the discovery of numerous germline and somatic mutations.

**Figure 1.**
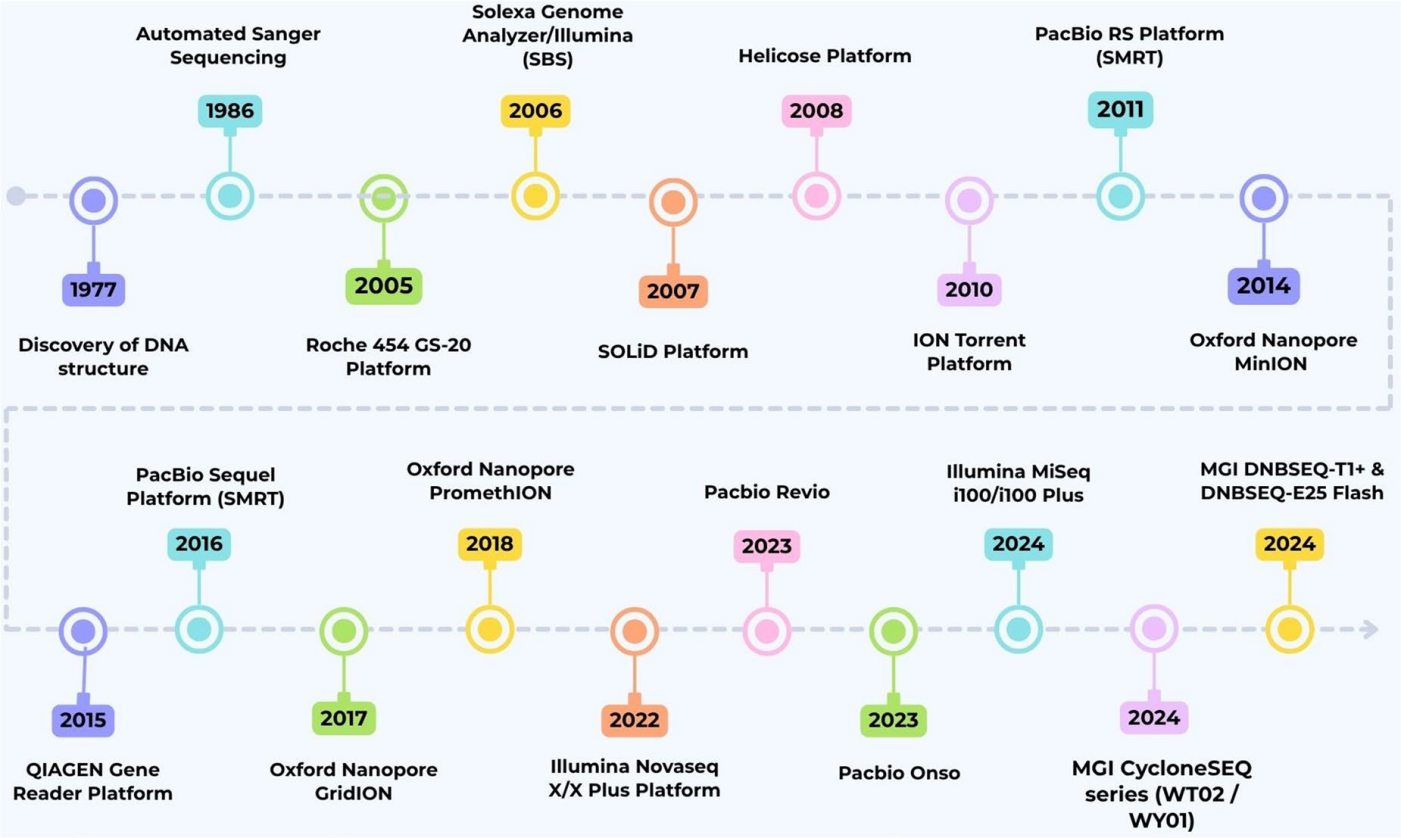
The chronological evolution of sequencing technologies. (Created using Canva).

The advent of TGS, or Long Read Sequencing, has further advanced sequencing capabilities by providing reads exceeding 10 kb in length. This improvement over short-read technologies enhances the assembly accuracy and enables better detection of structural variants. Platforms such as nanopore sequencing and PacBio SMRT sequencing offer cost-effective solutions with improved accuracy and promise for further genomic research and clinical applications. In Figure 1, we illustrated the major advancements of sequencing technologies over time, portraying the progression from first to third-generation sequencing.

### 2.2. Microarray technology

Microarray technology, also known as DNA chips or gene chips, is a high-throughput method that facilitates the concurrent measurement of gene expression across thousands of genes, single nucleotide polymorphisms (SNPs), copy number variations, and loss of heterozygosity throughout the genome, in contrast to NGS, which directly sequences DNA or RNA. This method employs microscopic slides with predetermined DNA sequences as its basis [11]. The detection of single nucleotide polymorphisms (SNPs), drug discovery, disease diagnostics, and personalized medicine are all significantly influenced by microarray technology. Perou and colleagues achieved a significant breakthrough through a gene expression microarray study, resulting in a redefinition of the molecular classification of breast cancer into five subtypes: luminal A, luminal B, basal-like, HER-2, and normal-breast-like [12]. This discovery establishes the groundwork for the advancement of genomic testing for early-stage breast cancer patients, such as Oncotype and Mammaprint, which assist in predicting the likelihood of breast cancer recurrence [13]. The Comparative Genomic Hybridization (CGH) approach, another form of microarray is utilized for identifying copy number variants, including chromosomal abnormalities, including deletions, additions, and ploidy variations. While array CGH, an advanced high-resolution technique, examines two variably fluorescently tagged test and reference samples across several loci in parallel, at higher resolution [14].

Microarray-based single-nucleotide polymorphisms (SNP) genotyping represents another type of microarray platform, designed to analyse the SNPs which are prevalent genetic variants uniformly dispersed throughout the human genome. These arrays comprise probes capable of differentiating between the two potential alleles at each SNP locus, facilitating the identification of homozygous and heterozygous genotypes. SNP arrays, due to the abundance and stability of SNPs, offer a robust basis for high-resolution, genome-wide screening, rendering them crucial in detecting genetic abnormalities. Uniquely, SNP arrays can simultaneously assess copy number alterations (gains or losses of DNA) and allelic variations (the loss of a specific allele) [15]. Additionally, the application of modern, high-throughput tissue microarrays (TMAs), which utilize tissue-based techniques such as immunohistochemistry (IHC), multiplex immunofluorescence, and fluorescent *in situ* hybridization (FISH), allows simultaneous analysis of a multiple number of tissues blocks [16]. This technology offers an economical approach for assessing several tissue samples and is time efficient in detecting cancer biomarkers. TMA block can be executed by punching tissue cores from designated areas of each block and organizing the punched cores into a recipient block, rather than individually sectioning each block, which is a labour-intensive procedure [17]. Tissue microarrays have been utilized in breast cancer research for biomarker development and validation, examination of tumour biology (including epithelial–mesenchymal transition), and assessment of the tumour microenvironment among various patient populations [18–20].

In the following section, we transition our focus to FDA-approved NGS testing in the domains of breast cancer detection, treatment response prediction, and genomic profiling. We will examine the practical applications of these technologies and their integration into clinical practice, elucidating their role in enhancing precision medicine for patients with breast cancer.

## 3. FDA-approved NGS tests

The United States Food and Drug Administration (FDA) has endorsed several NGS-based tumour profiling assays to characterize the molecular profile of solid malignant neoplasms [21,22]. Most assays employ a hybridization capture-based sequencing approach using various Illumina sequencing platforms.

In 2017, NGS-based MSK-IMPACT and FoundationOne CDx tests were the first to receive FDA approval. The MSK-IMPACT assay consists of a broad gene panel spanning all protein-coding exons of 486 genes, including all actionable genes. This test can detect Single Nucleotide Variations (SNVs), small insertions and deletions (indels), copy number alterations (CNAs), microsatellite instability (MSI) and selected gene rearrangements [23]. In the MSK-IMPACT landmark analysis published in *Nature Medicine,* genomic sequencing of tumours from 10,366 metastatic cancer patients revealed at least one actionable alteration in approximately 37% of the patients, suggesting that treatments targeting these mutations were either offered as part of routine care or in clinical trials [23]. This work has later been recognized as one of the seminal contributions in NGS research, leveraging the unmet needs of precision oncology.

Second, Omics Core is the first whole exome sequencing-based test for tumour mutation burden (TMB) approved by the FDA [24]. This test analyzes 19,396 whole exome protein-coding genes to assess total TMB using tumour-normal profiling, which allows an accurate assessment of TMB. In addition, this assay can report SNVs and INDELs across 648 cancer-associated genes with deep sequencing of ≥ 500X target coverage [24].

The PGDx elio^™^ tissue complete assay is a broad multigene panel comprising 521 cancer-related genes. In addition to detecting SNVs and small INDELs, this assay was able to assess *ERBB2* gene amplification, *ALK*, *RET*, *NTRK2*, and *NTRK3* translocations, MSI, and TMB. Utilizing hybrid-capture sequencing, this test can be performed using FFPE tumour tissues from patients with solid malignant neoplasms [25].

The xT CDx assay, which was recently approved by the FDA in 2023, is specifically designed with a broad target number of genes (648 genes) to identify INDELs, SNVs, and multiple nucleotide variations (MNVs). In addition, this test can also evaluate MSI status. This robust assay employs whole-genome shotgun library construction and hybridization-based capture sequencing technology, with the libraries being sequenced to a high read depth on Illumina NovaSeq 6000 sequencers. Apart from being approved as a companion diagnostic test for colorectal cancer patients, this test also serves for molecular profiling, which is applicable for all solid tumours [26].

The Guardant360 CDx test is the first FDA-approved liquid biopsy test for genomic testing that determines circulating cell-free DNA (cfDNA) from the plasma of peripheral whole blood. Guardant360 CDx utilizes targeted high-throughput hybridization-based capture technology to identify SNVs and indels across 55 genes, as well as copy number amplifications (CNAs) in four genes and fusions in two genes. This test served dual purposes: to profile tumour mutations in patients with any solid malignant neoplasm and as a companion diagnostic (CDx) test for non-small cell lung cancer with *EGFR, ERRB2, KRAS* mutations, and advanced breast cancer with *ESR1* mutations who may benefit from targeted therapy. It is the only FDA-authorized CDx for targeted breast cancer therapy in advanced/metastatic cases [27].

The FoundationOne Liquid CDx (F1LCDx) assay has a robust ability to interrogate 324 target genes using cfDNA extracted from plasma samples. This assay revealed SNVs and indels across 311 genes, CNAs in three genes (*BRCA1, BRCA2, ERBB2*), and gene rearrangements in eight genes (*ALK, BRCA1, BRCA2, NTRK1, NTRK2, NTRK3*). It serves as a CDx and a tumour profiling test for patients with solid malignant neoplasms. The CDx was recommended for four cancer types: non-small cell lung cancer, prostate cancer, breast cancer (*PIK3CA* mutations), colorectal cancer, and solid tumour with *NTRK1/2/3* fusions, which may benefit from the approved targeted therapies [28].

FoundationOne^®^CDx (F1CDx) comprises a gene panel spanning 324 cancer-associated genes for the identification of substitutions, indels, and CNAs in 16 genes, gene rearrangements, MSI, and TMB in FFPE tissue samples. As a CDx, this assay can identify patients with non-small cell lung cancer, melanoma, breast cancer, colorectal cancer, ovarian cancer, cholangiocarcinoma, prostate cancer, and solid tumours, who may benefit from the respective targeted therapies. Sequencing was performed based on hybrid-capture technology and in-depth sequencing, with an average coverage of >500X [29].

In conclusion, the landscape of cancer diagnostics and personalized medicine has been significantly transformed by the advent of FDA-approved Next-Generation Sequencing (NGS) tests. These assays offer a comprehensive molecular profiling of solid malignant neoplasms, enabling clinicians to tailor treatment strategies based on individual genomic profiles. From the pioneering MSK-IMPACT and FoundationOne CDx tests to the latest advancements such as the xT CDx assay and Guardant360 CDx test, each FDA-approved NGS test brings unique capabilities for detecting actionable alterations, assessing TMB, and guiding targeted therapy selection.

In the following section, we discuss the clinical applications of NGS in breast cancer. We will explore how these cutting-edge technologies are revolutionizing clinical practice, enhancing precision oncology, and improving outcomes in patients with breast cancer.

## 4. Clinical applications of NGS

### 4.1. Treatment guidance

Precision medicine in breast cancer tailors’ treatment to patients based on genetic makeup, tumour traits, and other factors. Using advanced technologies, such as genomic sequencing, clinicians can identify genetic alterations to guide therapy. This approach optimizes outcomes by selecting personalized treatments while minimizing side effects [30].

Kawaji et al. used NGS to characterize the genomic profiles of 115 advanced and metastatic breast cancer tissue samples. This analysis allowed the identification of actionable alterations, such as short variants in *TP53, PIK3CA, GATA3, PTEN,* and structural variants in *ERBB2, MYC, RAD21,* and *CCND1*. Subsequently, the researcher assessed whether the variants indicated the use of drugs based on medical evidence. The results allow the identification of patients who will benefit from poly ADP-ribose polymerase (PARP) inhibitors and DNA-damaging drugs and those with alterations that suppress *BRCA1/2* function. In addition, patients with MSI should be treated with immune checkpoint inhibitors. NGS was the initial step in establishing this precision oncology system, which will provide guidance for oncologists [31].

NGS demonstrated excellent sensitivity and specificity in quantifying HER2 amplification compared with IHC and FISH, achieving an area under the curve of 0.990 (95% CI: 0.982–0.999). NGS identified cases with HER2-negative status using IHC/FISH, with a specificity of 97.8%. Conversely, it can identify HER2-positive status using IHC/FISH, with a specificity of 99.8%. This high sensitivity and specificity will allow the identification of patients with low HER2 levels who will benefit from subsequent-line trastuzumab deruxtecan therapy [32].

#### 4.1.1. Prediction of chemotherapy response

Targeted DNA sequencing of 56 treatment-naïve TNBC samples showed that most mutations in this group of tumours appeared in *TP53*, *TTN*, and *PIK3CA.* The frequency of mutations was comparable between individuals who responded to treatment and those who did not (9 vs. 8 mutations). Notably, *PIK3CA* mutations were only detected in patients with intact *BRCA1* function. Samples from patients who experienced disease recurrence exhibited a higher rate of CNAs, with amplifications in *TTK* and *TP53BP2* correlated with a poorer response to chemotherapy [33].

#### 4.1.2. Resistance to herceptin and tyrosine kinase inhibitors (TKI)

Whole-genome sequencing was used to determine resistance to trastuzumab and TKIs. Comprehensive analysis showed that the frequency of C > T mutations was the highest in Herceptin-insensitive (HI) patients. MSI was not detected in trastuzumab-sensitive patients. Significant differences in the transition-transversion (TiTv) mutation ratios were observed between the trastuzeptin-insensitive and verification groups. In the TKI-insensitive (TI) group, C > T mutations were the most prevalent, differing from those in the TKI-sensitive (TE) group. The TE group included two patients with MSI-H. Significant differences in TiTv ratios were found between the TI and TE groups. Mutations in APOB may confer resistance to trastuzumab and TKIs, potentially leading to increased immune infiltration [34].

#### 4.1.3. Resistance to cyclin-dependent kinase 4/6 inhibitors (CDK4/6)

Using MSK-IMPACT, Li et al. found that loss of *RB1* and *FAT1* promotes resistance to CDK4/6 inhibitors in ER+/HER2- breast cancers before exposure to the drug. Progression-free survival (PFS) was significantly reduced in a limited subset of patients whose tumours harboured deletion of *FAT1* (the median PFS was 2.4 months (95% CI: 2.0, not reached) versus 10.1 months (95% CI: 8.7, 12.2]) or *RB1* (median PFS: 3.6 months, (95% CI: 2.2, not reached) versus 10.1 months (95% CI: 8.7, 12.4). In contrast, CDK4/6-treated patients whose tumours showed functional alterations in *CCND1* amplification, *PIK3CA* mutations, and *ESR1* mutations showed no response to the drug given [35].

Wander et al. performed whole-exome sequencing of biopsy samples from 58 patients with ER+ HR+/HER2− metastatic breast cancer treated with CDK4/6 inhibitors. The biopsy samples were identified as sensitive or resistant (intrinsic/acquired). Approximately 66% of resistant tumours showed enrichment of biallelic *RB1* disruption, *AKT1, RAS, AURKA, CCNE2, ERBB2,* and *FGFR2* alterations, and loss of oestrogen receptor expression. Biallelic *RB1* disruption was exclusively observed in resistant tumours. Additionally, exome sequencing in matched pre- and post-treatment samples from seven individuals who were resistant to CDK4/6 inhibitors showed that biallelic *RB1* and *AKT1* activation occurred concurrently, depicting clonal structure and dynamics [36]. These studies have shown that diverse genetic landscapes are associated with CDK4/6i resistance, which potentially affects clinical outcomes.

O’Leary et al. analysed ctDNA from 195 participants recruited in the PALOMA-3 study using paired whole-exome sequencing (*n* = 14) and targeted sequencing (*n* = 184). Patients receiving palbociclib plus fulvestrant possessed new driver mutations, *PIK3CA*, and *ESR1* mutations, which were enriched at the end of treatment. Tumours harbouring the *ESR1 Y537S* variant are likely to be more resistant to fulvestrant in this combined therapy. Interestingly, *RB1* mutations were found exclusively in the palbociclib plus fulvestrant group, although they were present in a small subset of the patients (6/127, 4.7%, *p* = 0.041). Driver gene mutations were prevalent in individuals who responded later to treatment but uncommon in patients who had an early response to palbociclib plus fulvestrant, which suggests that other mechanisms of resistance may predominate in the early stages of the tumour’s evolution [37].

Recently, Andre et al. (2023) assessed the response to CDK4/6i in advanced breast cancer patients by combining the baseline ctDNA dataset of 1530 patients who participated in the MONALEESA-2, 3, and 7 trials. A targeted NGS panel was used (577 genes). The analysis revealed that patients with *ERBB2*, *FAT3*, *FRS2*, *MDM2*, *SFRP1*, and *ZNF217* were sensitive to ribociclib. In contrast, patients harbouring alterations in *ANO1*, *CDKN2A*/*2B*/*2C*, and *RB1* were unresponsive to ribociclib. These genomic alterations are potential biomarkers of ribociclib responsiveness and resistance [38]. In Table 1, we summarize the molecular alterations associated with CDK4/6i resistance.

**Table 1.** Genomic alterations associated with resistance to CDK4/6 inhibitors.

Genes	Alterations	Sample size/Molecular subtype	Types of samples	NGS methods	Reference
*RB1, FAT1*	Loss	348/ER+/HER2- breast cancers	Formalin-fixed paraffin embedded tumour biopsy samples /or mononuclear cells from peripheral blood.	Targeted sequencingMSK-IMPACT	[25]
*RB1, ER*	Loss	58 ER+ HR+/HER2− metastatic breast cancer	Snap frozen metastatic core biopsies, FFPE blocks of primary tumour samples, whole blood	Whole exome sequencing	[26]
*RAS, ERBB2*	Activating mutations				
*AKT1, FGFR2*	Activating mutations and/or Amplifications				
*AURKA, CCNE2*	Amplifications				
*RB1*	Loss	195 patients from PALOMA study	Plasma	ctDNA whole exome sequencing and targeted sequencing (custom panel)	[27]
*ZNF703, FGFR1, CDK4, MDM2, FRS2*	Amplifications				
*CDKN2B, RB1, PTEN*	Loss				
*FAT1*	Mutations/loss				
*UGT1A1, NRG1, ABCC3, SPOP, TSC2, NFKBIA*	Mutations				
Heterozygous *RB1*	Loss				
*CDK4*	Copy number gain				
Homozygous *RB1 loss* and *FAT1*	Loss				
*ERBB2, FAT3, FRS2, MDM2, SFRP1,* and *ZNF217*	Mutations	1530 patients who participated in MONALEESA-2,3,7 trials	Plasma	ctDNA targeted sequencing	[28]
*ANO1, CDKN2A/2B/2C,* and *RB1*	Mutations/loss				

From predicting treatment responses to identifying mechanisms of resistance, NGS-based approaches provide invaluable insights that shape therapeutic strategies and improve patient outcomes. As our understanding of the genomic landscape of breast cancer continues to evolve, NGS remains a vital tool for unravelling the complexities of the disease. Next, we shed light on the use of NGS to understand the early onset of breast cancer.

## 5. NGS in early onset breast cancer (EOBC)

NGS technology has shaped the profile of breast cancer arising at a young age, providing adequate information regarding genomic alterations that can be used to customize therapy for this group of patients.

Targeted NGS used in a large cohort of 1276 pre-menopausal HR+/HER2- patients revealed that younger patients (<40 years, *n* = 359) harboured lower rates of mutations in *PIK3CA* (32% versus 47%), *CDH1* (3% versus 9%), and *MAP3K1* (7% versus 12%), and higher mutation rates in *GATA3* (19% versus 16%) and *TP53* (7% versus 3%, *q* < 0.010). CNAs were also higher than in their older counterparts (47% versus 26%). *PIK3CA* mutations, an oncogenic driver gene that significantly differed in prognosis between women under 40 and those over 40 years (HR 1.78, 95% CI 1.08–2.92), with *p* < 0.002 [39].

Another study used whole-exome sequencing on a cohort of 187 young Korean breast cancer patients (88.2% were pre-menopausal), and the results were compared with TCGA cohort’s whole-exome sequencing data (72.3% were post-menopausal). Younger patients had higher enrichment of somatic changes in *TP53* (47.9% vs. 32%), *PIK3CA* (28.5% vs. 32%), and *GATA3* (12.4% vs. 9.1%). An increased prevalence of *BRCA1/2* germline mutations was also observed in younger patients, which affected 13.7% of young patients (age ≤ 40 years) but only 4.8% of the intermediate age group (age > 40 years) (*p* = 0.08) and 3.4% of the TCGA cohort of the older age group (age > 60 years) (*p* = 6.85e-05) [40].

Andrikopoluou et al. characterized the mutational landscape of young breast cancer patients by performing NGS in 32 young patients (<40 years) and 90 older patients with breast cancer. The most frequent germline pathogenic mutations in the young patients were *CHEK2* (12%) and *BRCA1* (8%). However, the older counterparts harboured less frequent germline mutations in *BRCA1* (3%), *ATM* (3%), and *CHEK2* (3%). However, the study concluded that there was no significant difference in the genomic profile of breast cancer developed early from that developed late in life [41].

Ma et al. also contributed by analysing published multi-omics data of young (≤39 years) and elderly (aged ≥65 years) TNBC. Somatic mutations in *PIK3CA* (young vs. intermediate vs. elderly patients): (0.0% vs. 17.4% vs. 31.0% [*p* =.004]), *KMT2D* (4.0% vs. 2.4% vs. 11.9% [*p* = .020]), *ERBB2* (0.0% vs. 0.9% vs. 7.1% [*p* = .037]), *ERBB3* (0.0% vs. 0.5% vs. 7.1% [*p* = .015]), and *NCOR2* (0.0% vs. 0.9% vs. 7.1% [*p* = .035]) were significantly more common in older patients. In contrast, the young counterparts harboured more *COL3A1* mutations (12.0% vs. 4.7% vs. 0.0% [*p* = .005]) [42]. Waks et al. performed whole-exome sequencing (WES) to identify somatic and germline mutations in 92 young women with breast cancer who were diagnosed ≤35 years of age. Data for the old comparison group were obtained from TCGA database ≥45 years (range, 45–90 years). Young women harboured higher *GATA3* (43% vs. 12%) and *ARID1A* mutations (18% vs. 2%) and fewer *PIK3CA* mutations (14% vs. 38%) (*q* < 0.05) than older women [43]. In Table 2, we summarized the genomic alterations in EOBC detected by NGS.

**Table 2.** Genomic alterations observed in early onset breast cancer using NGS technology.

Genes	Alterations	Populations	Types of samples	NGS method	References
*GATA3, TP53*	Mutations	1276 HR+/HER2- early breast cancer patients (EBC) (<40 years)	FFPE tissues	Targeted sequencing (custom gene panel)	[29]
*TP53, PIK3CA, GATA3*	Mutations	187 premenopausal young Korean breast cancer women (≤40 years)	Fresh tissues and whole blood	Whole exome sequencing and RNA sequencing	[30]
*PIK3CA, TP53, BRCA2, PTEN*	Mutations	32 young breast cancer women (<40 years)	FFPE tissues and whole blood	Targeted sequencing	[31]
DNA repair genes, including *ATR, BAP1, ERCC6, FANCD2, FANCL, MLH1, MUTYH, PALB2, POLD1, POLE, RAD9A, RAD51, TP53*	Single nucleotide variations (SNVs)				
*CCND1, ERBB2, RPS6KB1, ZNF703*	Amplifications				
*GATA3, ARID1A*	Mutations	92 young women from from Young Women’s Breast Cancer Study cohort (YWBC)	FFPE tissues and peripheral blood mononuclear cells	Whole exome sequencing	[33]

## 6. Refining the molecular profile of well-established molecular subtypes of breast cancer

### 6.1. Characterizing HER-2 low breast cancer subtype

Earlier studies showed that there are two subgroups of HER2-negative breast cancers: ‘HER2-low breast cancer’, which is defined as breast cancer with lower HER2 expression when tested by IHC assay (score 1+) or IHC 2+ without gene amplification tested by ISH assay; and ‘HER2-zero breast cancer’, which is defined as a breast cancer without HER2 overexpression when tested by IHC assay (score 0). In recent years, the concept of HER2-low has emerged as a new clinically relevant category. It is crucial to note that HER2-low is not a molecular category in its own right, but rather an IHC-based classification. The success of antibody–drug conjugates (ADCs) like trastuzumab deruxtecan, which exhibit efficacy in tumours with minimal levels of HER2 protein expression [44,45] has been the driving force behind its recognition. This paradigm shift has established a therapeutic opportunity for patients who were previously classified as HER2-negative, further emphasizing the extent of HER2 expression in breast cancer.

Although NGS does not directly categorize HER2-low, it offers supplementary molecular insights that can enhance comprehension of HER2 biology. Varying mutations, amplifications, and structural variants can be detected by NGS, regardless of IHC status. These modifications may contribute to the explanation of the diverse clinical responses observed in HER2-low patients and can affect their response or resistance to HER2-targeted therapies. For example, an extensive genomic profiling has revealed that HER2-low and HER2-0 tumours exhibit largely analogous mutational landscapes, with no significant disparities in tumour mutational burden; however, HER2-low cancers may display slightly elevated *ERBB2* copy number alterations [46]. A recent study indicated that amplifications in cell-cycle genes may contribute to primary resistance against HER2-targeted antibody-drug conjugates (ADCs), whereas co-amplification of *ERBB2* and *CDK12* was associated with improved progression-free survival, implying that next-generation sequencing (NGS) can aid in identifying genomic predictors of ADC efficacy [47].

Additionally, HER2 mutations alone can drive response to targeted therapy, even in tumours without HER2 amplification or overexpression by standard IHC/ISH tests, that would otherwise be missed. The SGNTUC-019 phase II basket trial revealed that patients with HER2-mutated breast cancer, including those categorized as HER2-low or HER2-0 by IHC, exhibited a response to the combination of tucatinib and trastuzumab [48]. The data indicate that HER2 mutations can activate the HER2 pathway in tumours lacking protein overexpression, suggesting that these patients may also benefit from HER2-targeted therapy. Thus, NGS offers an adjunctive method to conventional pathology, facilitating the detection of individuals with HER2-low tumours who may benefit from targeted therapy, even with a negative or low IHC/ISH status. Furthermore, HER2-low tumours exhibit co-occurring mutations in significant oncogenic pathways such as *PI3K/AKT/mTOR (PIK3CA)* and tumour suppressor genes like TP53. These mutations may impact prognosis, therapeutic resistance, and tumour behaviour independently [49].

Another significant contribution of NGS lies in elucidating tumour heterogeneity and clonal evolution. The prognostic utility of IHC alone may be limited by intratumoral heterogeneity in HER2 expression and the presence of other genetic factors. In fact, new genomic research highlights that HER2-low is a part of a spectrum of HER2 expression rather than a biologically separate subtype, where ADCs might still be therapeutically beneficial even in the absence of gene amplification [50].

Herein, we summarized several NGS-based studies that compared the mutation profiles between HER2-low and HER2-0 breast cancer patients, with one study showing no significant difference [46], whereas a few studies showed subtle variations in the genomic profile of HER2-low and HER2-0 breast cancer patients [51,52].

Tarantino et al. compared HER2-low (*n* = 487, 47%) and HER2-0 (*n* = 552, 53%) genomic profiles utilizing targeted sequencing, results revealed no significant differences. Common oncogenic mutations were detected in both groups; which are *TP53, PIK3CA, CDH1, GATA3* and *ESR1.* However, CNV analysis showed a difference in the distribution of CNVs between the two entities, as an example of *FGFR1* amplification (12.3% HER2-low, 9.8% HER2-0). After correction for multiple tests, no genomic differences were observed [46].

In contrast, other studies have demonstrated considerable variations in the genetic landscape between HER2-low and HER2-0 cancers when the ER/HR expression status is considered. Tsai et al. analysed the genomic profiles of 615 HER2- patients using the OncomineTM (TMO) Comprehensive Assay, which spans 161 genes and found significant differences in molecular profiles between HER2-low and HER2-0 tumours. Comparative analysis of single nucleotide alterations (SNAs) between the two groups showed that *PIK3CA* was significantly enriched in HER2-low (17.62%; 65/369) compared to HER2-0 (9.35%; 23/246) (*p* = 0.006). In contrast, CNAs of *PIK3CA* (2.85%), *CCND3* (2.44%), and *CCND2* (2.85%) were commonly found in the HER2-0 rather than in the HER2-low subgroup (*p* < 0.05) [51].

Another study reported marginal changes in the mutational profiles of HER2-low and HER2-0 breast cancer patients. This study analysed NGS data from 556 subcohort tumours from the GeparSepto clinical trial (Loibl et al. 2019). HER2-0 had a significantly higher frequency of *TP53* mutations (24.8% vs. 16.3%; *p* = 0.018), whereas *PIK3CA* mutations were significantly enriched in HER2-low-positive tumours (44.0% vs. 33.4%; *p* = 0.012). However, upon stratification based on hormone receptor status, patients with hormone receptor-positive tumours harboured significantly higher rates of *TP53* mutations in the HER2-0 as compared to the HER2-low-positive subgroups (38.0% vs. 25.0%; *p* = 0.0078). In contrast, no significant differences were observed in *PIK3CA* or *TP53* mutation rates between HER2-low and HER2-low-positive subgroups among patients with hormone receptor-negative tumours [52].

Marra et al. (2023) revealed no significant changes in mutational signatures and TMB of HER2-low patients in general, even after correcting for HR expression among 3608 HER2- breast cancer patients who underwent targeted sequencing with the MSK-IMPACT assay. However, the analysis revealed marginal differences in the prevalence of gene alterations upon stratifying the HER2-0 and HER2-low subgroups according to hormone receptor status. Patients who were hormone receptor-positive harboured predominant *TP53* (33% vs. 25%; OR 1.49; 95% CI 1.25–1.78, *q* < 0.001) mutations in HER2-0 in comparison to HER2-low subgroups in general and metastatic contexts. The primary/early-stage tumours with hormone receptor-positive tumours did not exhibit any significant differences between the HER2-0 and HER2-low subgroups. No differences were observed between hormone receptor-negative patients in both subgroups [53].

An interesting contribution was made by Dai et al. who pooled and analysed the multi-omics data of 434 HER2-low breast cancer patients who underwent WES, OncoScan CNA assay, RNA sequencing, TMT quantitative proteomics, and metabolomics. These multi-omics data were analysed at the genomic, transcriptomic, proteomic, and metabolomic levels. This study showed that the differences in HER2-low and HER2-0 cancers with HR-positive status were comparatively minor at the transcriptome, proteomic, and metabolomic levels. The molecular features of HER2-low tumours were more profound in HR- HER2-low as compared of HER2-0 tumours. Patients with non-basal-like disease were more enriched in HR the HER2-low subgroups rather than HR- HER2-0 subgroups (30.3% vs. 3.7%, *p* = 0.005) and were shown to have a greater resemblance to HER2-positive tumours [54]. In Table 3, here, we summarized the genomic signatures related to HER2-low breast cancer.

**Table 3.** Genomic alterations associated with HER-2 low breast cancer subtype.

Genes	Alterations	Populations	Types of samples	NGS Method	References
*PIK3CA* and *ERBB2*	Mutations	615 HER2 patients	FFPE tissues	Targeted sequencing (OncomineTM (TMO) Comprehensive Assay)	[35]
*PIK3CA*	Mutations	556 tumours of sub-cohorts from the GeparSepto clinical trial	FFPE tissues and blood	Targeted sequencing	[36]
*TP53,* Transcription factors genes *(MYC,YAP1)* and DNA damage response genes *(FAM175A, BRCA2),* and *PIK3CA*	Mutations	3608 HER2-breast cancer patients	Tissue biopsies	Targeted sequencing (MSK-IMPACT)	[37]
*PIK3CA*	Mutations	434 HER2-low breast cancer patients	Fresh frozen tissues and peripheral blood cells	WES, OncoScan CNA assay, RNA sequencing, TMT quantitative proteomics and metabolomics	[38]
*FGFR4, PKT6, ERBB4*	Overexpression				
17q12	Gain/amplification				
17q11.2, 17q21.31	Loss/deletions				

From the above evidence, we may conclude that NGS plays a crucial role in refining our understanding of the genetic mock-up of complex tumours that lack specific targeted therapies. Given the increasing clinical significance of HER2-low, future research should assess the potential of NGS to complement IHC by identifying genomic signatures or biomarkers that are predictive of ADC benefit. This would represent a transition from protein-level classification to a more comprehensive molecular definition of HER2 biology.

## 7. The dynamic duo of circulating tumour DNA (ctDNA) and NGS

NGS holds significant utility in identifying cancer-specific mutations within circulating tumour DNA and is commonly referred to as liquid biopsy. This non-invasive technique serves as a valuable screening tool and aids in overcoming the challenges associated with procuring tissue biopsy samples [55]. ctDNA application extends across the entire spectrum of disease progression, encompassing early detection, evaluation of minimal residual disease following treatment, and monitoring of treatment response and resistance [56]. ctDNA is a fragment of double-stranded DNA that typically ranges from 160 to 180 bp. It is released from tumour cells into the bloodstream *via* processes such as necrosis and apoptosis. The quantity of ctDNA present in the peripheral blood of cancer patients offers valuable insights into the presence of cancer [57]. Earlier studies have demonstrated that ctDNA can be effectively employed to gain insights into the genomic landscape of metastatic breast cancer.

Molecular Barcode NGS (MB-NGS) was employed to detect mutated ctDNAs in breast cancer patients. An NGS tool was tailored with a panel of the 13 most commonly mutated genes in breast cancer. The results showed that among stage I and II breast cancer patients, 62% harboured 95 somatic mutations across 12 genes. Further exploration of the same somatic mutations in plasma samples collected from the same patients before surgery revealed that 16% carried ctDNA with precisely matching mutations [58].

Examination of the mutational profile of metastatic breast cancer in a larger cohort of patients (*n* = 255) by employing NGS on matched tissue and blood biopsies revealed that the genomic heterogeneity of metastatic breast cancer was prominently evident in circulating tumour DNA (ctDNA). The concordance between the tissue and blood biopsies was notably high, ranging from 79% to 91%. Notably, triple-negative tumours exhibited the highest mutant allele frequency compared with hormonal-positive and human epidermal growth factor-positive tumours (*p* < 0.05). The most prevalent mutations were *PIK3CA* (39.6%), *ESR1* (16.5%), and *ERBB2* (21.6%). These results suggest that ctDNA serves as a reliable source for evaluating the mutational landscape of metastatic breast cancer [59].

In terms of utilizing ctDNA to predict breast cancer progression, a previous study utilized ctDNA to understand the genomic profile behind the development of resistance in metastatic breast cancer patients and reported that besides the heterogeneity related to the molecular subtype of breast cancer, an additional layer of heterogeneity was added. Among patients with hormone receptor-positive breast cancer who experienced progression within 3 months of treatment, there was a notable increase in the frequency of *TERT* mutations. Additional relevant mutations included those in *FAT1* and *NOTCH4*. For those who had tumours that progressed between 3 and 6 months, the candidate mutations implicated were *PIK3CA, TP53, MLL3, ERBB2, NOTCH2*, and *ERS1*. These findings suggest that diverse mechanisms drive disease progression at different post-treatment intervals. Notably, the ctDNA *TP53* +* PIK3CA* mutation pattern in the COX model effectively predicted progression within 6 months [60].

Exploring ctDNA mutations that can be used in breast cancer therapeutic decision-making and patient prognosis. A study utilizing targeted NGS that spanned 1021 genes found that *PIK3CA* mutations were more prevalent in HER2+ tumours, correlating with shorter median progression-free survival and poorer overall survival. *TP53* mutations were associated with shorter overall survival (median 64 vs. 121 months, *p* = 0.006). ctDNA analysis revealed a higher frequency of *PIK3CA* mutations in HER2+ disease, indicating worse OS and serving as the exclusive mutation associated with shorter progression-free survival in a multivariate analysis of HER2+ patients treated with trastuzumab, suggesting reduced trastuzumab efficacy in these patients [35].

Employing targeted NGS for predicting recurrence in patients with TNBC with residual disease following neoadjuvant therapy can offer high specificity, but may potentially lack sensitivity. One study used the Oncomine Research Panel, which comprises 134 cancer genes. The researchers were able to detect mutations in ctDNA in four out of 13 patients. Although all four patients experienced recurrence, which reflects the specificity of the technology, nine cases were missed, which may reflect less sensitivity [61].

Another approach adopted by cancer researchers is the use of serial ctDNAs to monitor disease progression. In an earlier study, disease progression was monitored clinically and *via* imaging. ctDNA analysis revealed significant alterations in *TP53, PIK3CA, AR, FGFR1,* and *ESR1* between the baseline blood samples and the first point of progression. There was an increase in mutant allele frequency (MAF) and number of alterations (NOA) between the baseline and the first point of progression. Furthermore, compared to individuals without disease progression, patients who underwent ctDNA collection at the time of disease progression exhibited higher MAF and NOA [62].

Based on the evidence provided, it can be inferred that circulating tumour DNA (ctDNA) and NGS possess the potential to serve as valuable tools for the diagnosis, prediction, prognosis, and monitoring of breast cancer, despite the heterogeneity of the genomic profiles of this disease.

## 8. Single-cell sequencing in breast cancer as an emerging potential discovery tool

Cellular variation from one cell to another is a well-established characteristic of multicellular organisms, where tissues, even those that seem uniform, consist of diverse cell types. Recent advancements in single-cell analysis technologies have introduced an unparalleled level of understanding of cell diversity at the genomic, transcriptomic, proteomic, and metabolic levels. These innovations challenge oversimplified views of cell populations and offer a comprehensive understanding of disease mechanisms, such as clonal evolution within tumours and the influence of tumour microenvironments. Successful exploration of the complete complexity of cells in multicellular organisms requires the integration of improved experimental and computational methods [63]. The concept of single-cell RNA sequencing (scRNA-seq) was first described in 2009 by Tang et al. [64]. Single-cell analysis enriched breast cancer literature in various critical areas, which we highlighted in this section.

### 8.1. Transcriptomic heterogeneity of breast cancer

Through single-cell RNA-sequencing (scRNA-seq) analysis of more than 1500 cells derived from six primary TNBCs, significant variability in gene expression profiles among cells was observed. The different gene expression profiles were linked to clonality in the genomic CNAs. A subpopulation of cells showed the activation of pathways related to glycosphingolipid metabolism and innate immunity. This gene signature provides predictive value for long-term outcomes in patients with TNBC, where it is related to treatment resistance and metastasis [65].

A recent study revealed the heterogeneity of breast cancer by profiling the transcriptome of 35,276 individual cells derived from 32 cell lines. This study revealed significant heterogeneity in biomarker expression. This study linked the gene expression profile of each cell with large-scale *in vitro* drug screening to predict drug responses. The results showed that transcriptional heterogeneity allowed cells with varying drug sensitivities to coexist within the same population [66].

Further exploration of the heterogeneity of breast cancer through single-cell analysis will lead to the identification and understanding of the heterogeneity of recurrent breast cancer. The researcher distinguished nine intrinsic subtypes (ecotypes) by examining PD-L1/PD-L2 macrophage cells, which are related to the clinical outcome, cell surface expressed proteins, and mesenchymal cell components. Collectively, these factors provide insights into the anti-tumour immune mechanism. The nine types have distinct cellular components and clinical outcomes [67]. Using single-cell sequencing, researchers have differentiated carcinoma cells from noncancer cells based on CNVs. The majority of non-cancer cells identified were immune cells, comprising three distinct clusters: T lymphocytes, B lymphocytes, and macrophages. Both T lymphocytes and macrophages exhibit immunosuppressive traits, with T cells demonstrating either a regulatory or exhausted phenotype and macrophages displaying an M2 phenotype [68]. These findings underscore the wide-ranging intratumoral heterogeneity present in the breast cancer transcriptome that is influenced by both tumour cells and immune cells within the tumour microenvironment.

### 8.2. Metastasis

Using single-cell RNA sequencing, a recent study compared the transcriptomic profiles of primary tumours with lymph node metastasis. By isolating breast cancer stem cells (BCSCs) and investigating the CNVs of BCSCs, results revealed that it’s suggested BCSCs originated from normal breast cancer cells. As cancer grows, cells acquire more mutations, especially those linked to spreading to the lymph nodes. Metastatic cancer cells hinder the body’s ability to fight tumours by binding to receptors on immune cells that inhibit their function. This interaction creates an environment that supports tumour growth. NECTIN2-TIGIT interaction were identified between metastatic non-TNBC cells and T cells. It is noteworthy that these molecules were previously thought to be expressed by developed immune cells [69].

Another study utilizing single-cell sequencing technology confirmed a previous finding that the immune microenvironment in lymph node metastatic breast cancer shows a state of suppression in comparison to the primary tumour microenvironment. Various activities of T cells, including cytotoxicity and proliferation, were suppressed. The CD4+ CXCL13+ cells exhibited exhaustion. Meanwhile, LAMP3+ dendritic cells showed lower T cell activation capabilities. Pathways related to antigen presentation were also downregulated. All the evidence emphasizes the necessity of precision medicine for lymph node metastatic breast cancer [70].

Through scRNA-seq, it was found that mitochondrial oxidative phosphorylation (OXPHOS) is a pathway that is more active in micrometastases than in primary tumour cells, which tend to exhibit higher expression of genes associated with aerobic glycolysis. Blocking OXPHOS significantly reduced lung metastasis, indicating that OXPHOS plays a crucial role in promoting metastatic spread [59].

### 8.3. Prognosis

scRNA-seq of 6,311 T cells isolated from human breast cancers revealed significant diversity among the infiltrating T cell populations. In addition, breast cancer with high levels of tumour-infiltrating lymphocytes (TILs) contains CD8+ T cells, showing characteristics of tissue-resident memory T (TRM) cells. These CD8+ TRM cells express elevated levels of immune checkpoint molecules and effector proteins, which are associated with improved survival in early-stage TNBC. CD8+ TRM cells play a crucial role in breast cancer immune surveillance and are the primary targets for immune checkpoint therapy [71].

Single-cell transcriptome analysis showed that after anti-PD1 treatment, certain T-cell populations showed clonal expansion, regardless of the tumour subtype. This expansion was primarily observed in CD8+ and CD4+ T-cells. In treatment-naïve breast cancer, the presence of immuno-regulatory dendritic cells (PD-L1+), macrophages, and cancer cells expressing major histocompatibility complex class I/II correlates positively with T cell expansion. In contrast, the presence of undifferentiated pre-effector/memory T cells or inhibitory macrophages was inversely correlated with T-cell expansion. This finding may shed light on the heterogeneity of tumour cells in response to immunotherapy [72]. scRNA-seq is a potential discovery tool that offers a comprehensive understanding of breast cancer heterogeneity, enabling the exploration of the mechanisms of metastasis and drug resistance. It also facilitates the identification of markers to predict patient prognosis [73].

## 9. Conclusion

NGS has uncovered the genomic and transcriptomic diversity of breast cancer, revealing actionable alterations linked to chemotherapy response and resistance to therapies, such as trastuzumab, TKIs, and CDK4/6 inhibitors, which helps in treatment guidance for this particular group of patients. Moreover, NGS reveals the genomic alterations linked to early onset breast cancer, a high-risk patient demographic that often displays a greater incidence of triple-negative or HER2-positive subtypes, characterized by higher-grade malignancies and poor prognoses, thereby illustrating the distinct molecular landscape of this cohort. On the other hand, ctDNA shows promise in diagnosis, prediction, prognosis, and monitoring, despite the genomic heterogeneity of the disease. Single-cell analysis holds the potential for studying individual cell transcriptomes, but challenges such as high costs and low throughput must be addressed for widespread use. Entities such as HER2-low tumours pose ongoing research challenges. However, the integration of NGS analysis with the conventional pathology technique is a promising approach in HER2-low breast cancer, as it may uncover co-occurring genomic alterations that can enhance patient stratification and inform more precise targeted therapies.

## Data Availability

Data sharing is not applicable to this article, as no new data were created or analysed in this study.
